# Long-Pulse Gastric Electrical Stimulation Repairs Interstitial Cells of Cajal and Smooth Muscle Cells in the Gastric Antrum of Diabetic Rats

**DOI:** 10.1155/2018/6309157

**Published:** 2018-11-13

**Authors:** Yan Chen, Hongcai Wang, Hai Li, Shi Liu

**Affiliations:** ^1^Department of Gastroenterology, Binzhou Medical University Hospital, Binzhou, Shandong, China; ^2^Department of Neurology, Binzhou Medical University Hospital, Binzhou, Shandong, China; ^3^Division of Gastroenterology, Union Hospital, Tongji Medical College, Huazhong University of Science and Technology, Wuhan, Hubei, China

## Abstract

**Background/Aims:**

The damage of interstitial cells of Cajal and smooth muscle cells has far-reaching implications in the pathogenesis of gastroparesis in diabetic patients. Gastric electrical stimulation (GES) is an efficient therapy for gastric motility disorders, but the mechanisms of GES require clarification.

**Methods:**

Male rats were randomly divided into the control group, diabetic rat group (DM), diabetic rats with sham GES group (DM + SGES), and diabetic rats with different frequency GES group (DM + GES) (GES1: 5.5 cpm, 100 ms, 4 mA; GES2: 5.5 cpm, 300 ms, 4 mA; and GES3: 5.5 cpm, 550 ms, 2 mA). Gastric contractions were explored using the organ bath technique. The alterations of interstitial cells of Cajal, the SCF/c-kit pathway, and smooth muscle cells were also investigated.

**Results:**

(1) Gastric contractions were significantly improved in the DM + GES group compared with those in the DM group. (2) The damage of interstitial cells of Cajal was prevented in the DM + GES group in contrast to the DM group. Moreover, long-pulse GES increased the expression of the SCF/c-kit pathway. More proliferated interstitial cells of Cajal in muscle layers were observed obviously in the DM + GES group. (3) The number of smooth muscle cells in the DM group was not significantly decreased compared with that in the control group. However, ultrastructural changes were distinctly damaged in the DM group. The application of GES protected against the alteration of the ultrastructures of smooth muscle cells.

**Conclusions:**

Long-pulse GES improves gastric contraction possibly by enhancing the proliferation of interstitial cells of Cajal and restoring the injury of smooth muscle cells.

## 1. Introduction

Gastroparesis is a common medical condition in patients with long-standing type 1 and type 2 diabetes mellitus. Symptoms such as fullness, anorexia, nausea, bloating, vomiting, early satiety, and abdominal pain are most likely observed in diabetic patients. Conventional management, including the use of prokinetic and antiemetic agents, is not a permanent cure to reduce the symptoms. Thus, gastric electrical stimulation (GES) improves the symptoms of gastroparesis and might have clinical applications. The intrinsic molecular mechanism requires additional investigation and clarification.

The interstitial cells of Cajal (ICC) whose branches form gap junctions with smooth muscle cells (SMC) are the pacemaker cells for gastrointestinal movement. The decreased electrical slow waves attributed to the loss of ICC reduced the connection with SMC, resulting in delayed gastric peristalsis and irregular intestinal segmentation. Similarly, ICC has been found to be lost or diminished in experimental models and in diabetic patients [1]. ICC have been identified using a well-established molecule marker termed c-kit, and the incorporation with c-kit's ligand stem cell factor (SCF) is necessary for ICC to survive, develop, differentiate, and maintain function [2]. Previous studies have shown that the expression of SCF is decreased in diabetic animals and patients, accompanied with decreased expression of c-kit [3, 4]. Meanwhile, SCF expression was attributed to the function synthesis and secretion of SMC [5]. Therefore, whether SMC were involved in the damage of ICC in the development of diabetic gastroparesis remains puzzling.

Presently, the management of diabetic gastroparesis leaves something to be desired. It has demonstrated that GES could be potentially beneficial for improving gastric motility [6]. Numerous researchers have certified that the effects of GES were influenced by the parameters and that the mechanism of GES with different parameters was different [7, 8]. In particular, long-pulse GES, defined by a pulse width of 100–600 ms and similar to gastric pacing, was certified to ameliorate gastric slow waves, gastric emptying, and contraction of gastric antrum in various experimental models and in humans [9, 10]. Simultaneously, in a previous study, long-pulse GES was found to induce the regeneration of myenteric plexus synaptic vesicles [11]. However, the mechanisms of long-pulse GES for ameliorating gastric motility remained elusive.

Although some investigations demonstrated that the deficiency of ICC and SMC acting as a cofactor was the major etiologic factor in diabetic gastroparesis, the exploratory study for determining the effects of long-pulse GES on ICC and SMC is seldom performed. The aim of this study was to verify the effects of long-pulse GES on gastric motility and investigate the regulatory mechanisms in diabetic rats.

## 2. Materials and Methods

### 2.1. Animal Preparation

A total of 60 male Sprague-Dawley rats (weighing 160–200 g) were purchased from the Experiment Animal Center of Wuhan University and were raised in suitable laboratory conditions. The animals were treated humanely, and the experimental procedures were completed according to the recommendations in the Guide for the Care and Use of Laboratory Animals of the National Institutes of Health, following the ethical guidelines from the Animal Care and Use Committee of Tongji Medical College, Huazhong University of Science and Technology.

### 2.2. Electrode Implant

Given general anesthesia, 40 rats were submitted to surgery to implant electrodes imbedded in the stomach serosa 2 cm along the greater curvature above the pylorus. The ends of the wires were inserted subcutaneously through the abdominal wall, were passed out of the neck, and were connected to the physiological signal acquisition processing system.

### 2.3. Diabetic Model

After 2 weeks of recovering from surgery, diabetic models were established using an intraperitoneal streptozotocin injection. After one week, the blood glucose of the tail vein was measured and diabetes was confirmed twice with a blood glucose of >16.7 mmol/L.

### 2.4. Experimental Protocol

**Figure 1:**
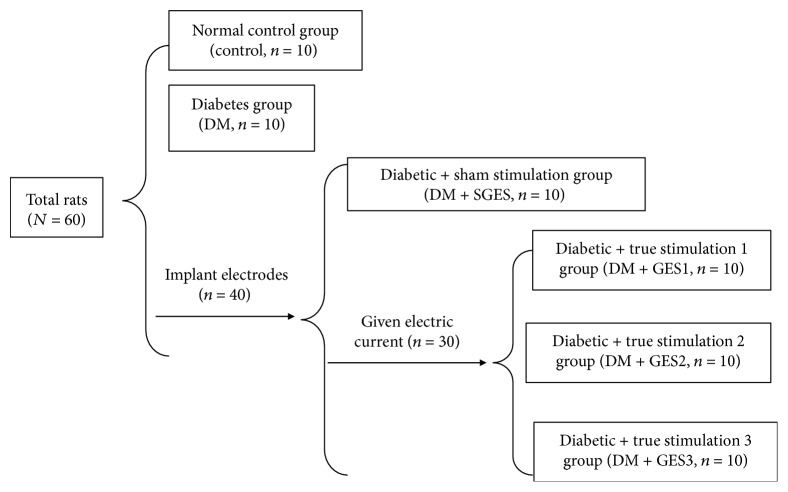
The flow chart of grouping.

The rats were divided into the normal control, diabetes, diabetic + sham stimulation (DM + SGES), and diabetic + true stimulation (DM + GES) groups (Figure 1). The DM + SGES group was not given electric current and was only connected to the instrument, while the DM + GES group was further divided into three groups (DM + GES1, DM + GES2, and DM + GES3) using different parameters [12, 13]: GES1: 5.5 cpm, 100 ms, 4 mA; GES2: 5.5 cpm, 300 ms, 4 mA; and GES3: 5.5 cpm, 550 ms, 2 mA. Stimulation was chronic, 30 min/day, for a total of 6 weeks. Circular muscle contraction was used to evaluate the gastric motility. Gastric antrum tissues were obtained for Western blotting, real-time polymerase chain reaction (RT-PCR), immunohistochemistry, and transmission electron microscopy.

### 2.5. Recording of Contractile Activity

The circular muscle strips of gastric antrum, 7 × 2 mm with the mucosal layer removed, were obtained. The strips were hung in the organ bath consisting of 37°C Krebs buffer, with continuous bubbles of 95% O_2_/5% CO_2_. The tension of the strips was monitored using isometric force transducers, which were connected to a chart recorder via an amplifier. The data of variation tendency were digitized and analyzed using the digital recording and analytical software (LabChart 7.0, ADInstruments, Castle Hill, Australia). A different concentration of acetylcholine (Ach 10^−7^ to 10^−3^ mol/L; Sigma, St. Louis, MO, USA) was added. A selective method for destroying ICC with methylene blue and illumination was used to assess contractions without ICC [14]. Cumulative concentration-response contractile curves were achieved. The area under the curve (AUC) revealed the contractile activity of circular muscle strips before and after removal of ICC. %Variation represented the contractile responses at each concentration of Ach contrasted with the baseline. %Variation was calculated as follows: %Variation = (AUC_Ach_ − AUC_baseline_)/AUC_baseline_ × 100%. %Decrease showed the variation of contractile responses before and after removal of ICC. %Decrease was calculated according to the following formula: %Decrease = %Variation_before ICC removed_–%Variation_after ICC removed_.

### 2.6. Western Blotting

Fresh-frozen specimens of distal stomach were crushed into cell suspension and homogenized in RIPA buffer, including a protease inhibitor. After centrifugation, the supernatants were collected as the total protein. A total of 60 mg of proteins was separated by 10% sodium dodecyl sulfate polyacrylamide gel electrophoresis and then was transferred to a PVDF membrane. These membranes were incubated with anti-c-kit antibody, anti-SCF antibody, anti-*α*-SMA antibody, and rabbit anti-mouse GAPDH antibody. Then, secondary antibody was offered. Signal detection was visualized using an enhanced chemiluminescent agent, and the blot was present and detected by autoradiography.

### 2.7. Real-Time PCR

Total RNA was extracted using the TRIzol reagent, and the purity and concentration of total RNA were obtained according to the absorbance readings using a spectrophotometer. RNA samples were reverse transcribed into cDNA according to the instructions. The specific primers used in the study are listed in Table 1. All PCR reactions were performed in a 10 *μ*L final volume, containing 5 *μ*L SYBR green/enzyme reaction mix, 50 nmol/*μ*L of each sense and antisense primer, and 1 *μ*L cDNA. The PCR conditions were 95°C for 10 min, followed by 35 cycles of 95°C for 15 s, and 60°C for 1 min. Real-time detection was conducted using ABI-StepOne Detection System (Applied Biosystems, Thermo Fisher Scientific Inc.). The melting curve analysis was performed by increasing the temperature by 1°C increments from 60°C to 95°C and measuring fluorescence at each temperature for a period of 10 s. The 2^−ΔΔCT^ method was adopted to quantify the relative change in gene expression.

### 2.8. Immunohistochemistry and Immunofluorescence Staining

The sample of gastric antrum was fixed in 4% paraformaldehyde and embedded in paraffin and cut into sections of 6 *μ*m. The sections were deparaffinized in xylene and hydrated in a graded ethanol solution. To inhibit the endogenous peroxidase activity, 3% hydrogen peroxide (H_2_O_2_) was used, the sections were microwaved (750 W) to expose the antigens, and nonspecific reaction was blocked by normal rabbit serum. The primary antibody c-kit and *α*-SMA were added. For immunohistochemical study, these sections were incubated in an orderly fashion with secondary antibodies and horseradish peroxidase- (HRP-) linked streptavidin. The target protein was displayed using a fresh 3,3′-diaminobenzidine (DAB) solution. After they were counterstained and dehydrated, the slides were mounted carefully with coverslips. For immunofluorescence staining, immunoreactivity was detected using Alexa Fluor 594 donkey anti-goat IgG and Alexa Fluor 488 donkey anti-mouse IgG. The sections were mounted using the fluorescence quenching agent and were examined using a microscope.

For whole-mount staining, gastric antrum was pinned to a Sylgard dish and fixed in Zamboni's fixative. With sharp dissection, a circular muscle layer including ICC-IM was obtained. The specific procedures were described in our previous study [15]. After washing with PBS containing 5% normal bovine serum and 0.3% Triton X-100 (PBST), primary antibody c-kit and Ki67 were added. The immunoreactivity was revealed by donkey anti-goat IgG-conjugated secondary antibody and donkey anti-rabbit IgG-conjugated secondary antibody, and the nuclei were stained with Hoechst. After they were unfolded on a slide, the tissues were checked under a confocal laser-scanning microscope.

### 2.9. Electron Microscopy

After the mucosa was removed, the specimens of antrum were immersed entirely in a fixative solution of 2.5% glutaraldehyde and 2% paraformaldehyde in 0.1 mol/L phosphate buffers. Then, the tissues were postfixed in 1% osmium tetroxide for 2 h, dehydrated using graded alcohol, clarified in propylene oxide, and embedded in EPON epoxy resin using flat molds. Ultrathin sections were obtained using an ultramicrotome, and they were stained with uranyl acetate and lead citrate before viewing the sections under a transmission electron microscope.

### 2.10. Statistical Analysis

All values were expressed as mean + SEM. The analysis of variance (ANOVA) by SPSS 17.0 was used to compare the differences among different groups. *P* < 0.05 was regarded as a statistically significant difference.

## 3. Results

### 3.1. GES Improved Mechanical Contraction Activities of Circular Muscle

**Figure 2:**
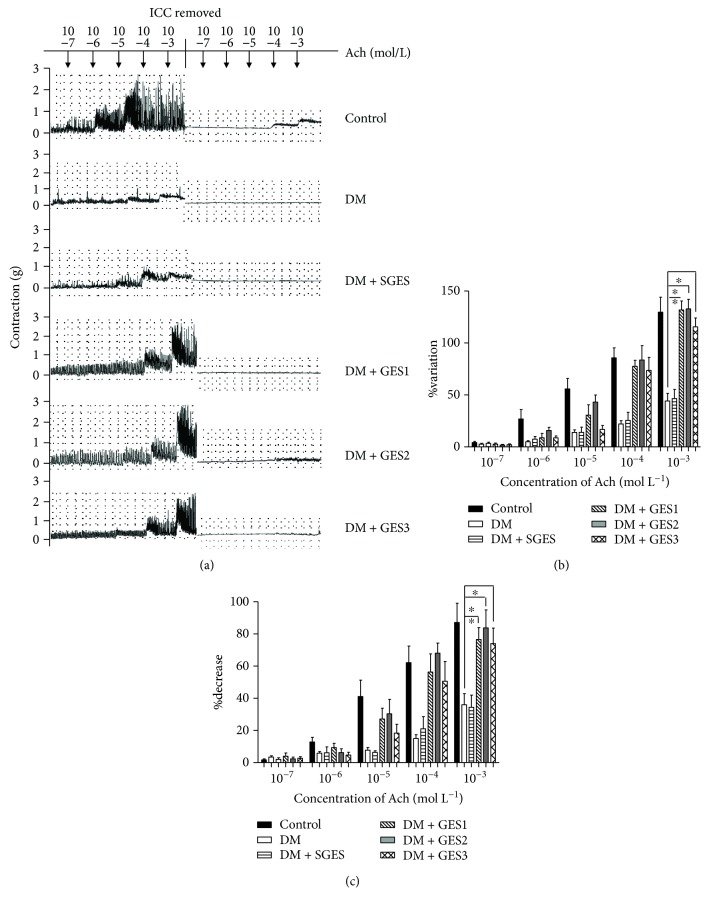
The contractile activity of gastric antrum circular muscle strips. (a) When the concentration of Ach was up to 10^−3^ mol/L, the gastric strips reached to the greatest degree contraction. (b, c) Both %Variation and %Decrease in the DM group were significantly decreased in contrast to those in the control group, while the contractions of antral muscle strips in diabetic rats with true GES were all obviously increased. ^∗^*P* < 0.05 compared with the DM group.

The concentration-dependent contraction of gastric antrum circular muscle strips was induced by Ach (Figure 2(a)). When the concentration of Ach was from 10^−7^ to 10^−3^ mol/L, the differences of contraction were found.

As shown in Figure 2(b), %Variation signified the contractions of gastric antrum circular muscle in the different groups. When Ach was up to 10^−3^ mol/L, %Variation in the DM group was significantly decreased compared to that in the control group (*P* = 0). There was no significant difference in %Variation between the DM and DM + SGES groups (*P* = 0.865), while increased %Variation represented contractions of antral circular muscle strips indicated in the DM + GES groups compared with the DM group (all *P* = 0).

To reveal the effects of interstitial cells of Cajal in the contractions, %Decrease was employed to evaluate the cell number of interstitial cells of Cajal (Figure 2(c)). As the concentration of Ach increased up to 10^−3^ mol/L, the %Decrease in the DM group was significantly reduced compared with that in the control group (*P* = 0.013). The %Decrease had no significant difference between the DM + SGES group and the DM group (*P* = 0.94). However, the %Decrease in the DM + GES group was significantly elevated in contrast to that in the DM group (*P* = 0.011, 0.003, and 0.034).

### 3.2. Increased Expressions of ICC and SCF/c-kit Signaling after GES Treatment

**Figure 3:**
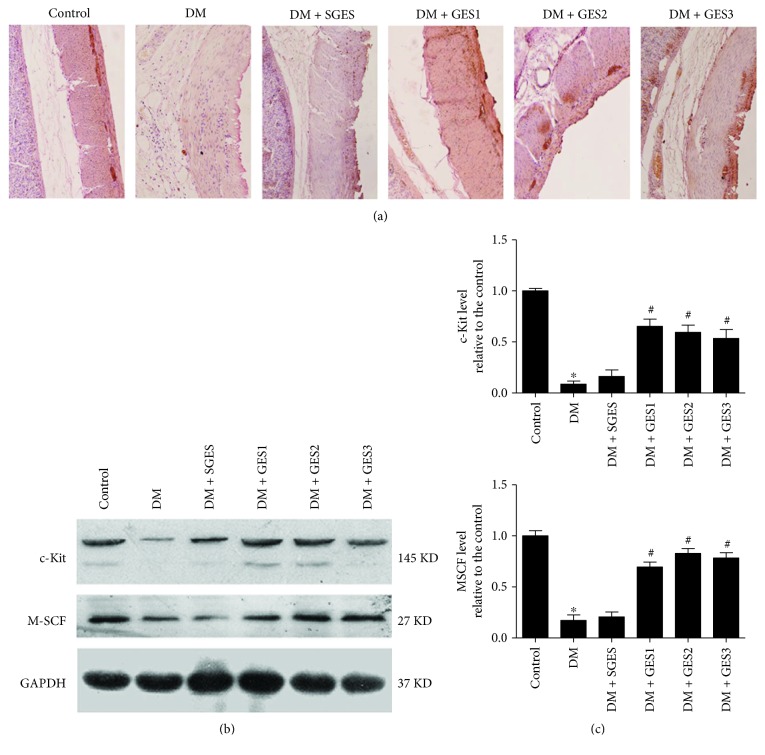
ICC and SCF/c-kit signaling expressed in each group. (a) Immunohistochemical technique showed that a large number of c-kit+ cells in the DM + GES groups were distributed in the intramuscular layer and the myenteric layer of the gastric antrum (200x). (b) Western blot study showed that after given long-pulse GES, the expressions of c-kit and SCF protein were significantly increased compared with those in the DM group. (c) RT-PCR showed that the expressions of c-kit and SCF mRNA in the DM + GES groups were significantly increased compared with those in the DM group. ^∗^*P* < 0.05 in the DM group compared with the control group. ^#^*P* < 0.05 in other groups compared with the DM group.

As shown in Figure 3(a), abundant c-kit+ cells were found in the intramuscular layer and the myenteric layer in the control group, while decreased c-kit+ cells were detected in the gastric wall of the DM and DM + SGES groups. Interestingly, treated with GES, the number of c-kit-positive cells was increased and distributed in the intramuscular and myenteric layers of gastric antrum.

According to Figure 3(b), both c-kit and M-SCF protein expressions in the DM group were significantly decreased compared with that in the control. There was no significant difference in the c-kit and M-SCF expressions between the DM group and DM + SGES group. Long-pulse GES with different parameters all enhanced the protein expressions of c-kit and M-SCF.

Consistently, as shown in Figure 3(c), the mRNA expressions of c-kit and M-SCF in the DM group were decreased compared with those in the control group (both *P* = 0). The mRNA expressions of c-kit and M-SCF were significantly elevated in the GES group in contrast to those in the DM group (c-kit: *P* = 0, 0.001, and 0.013; SCF: all *P* = 0).

### 3.3. Dependent on the Effects of GES, Proliferation of ICC-IM Was Involved

**Figure 4:**
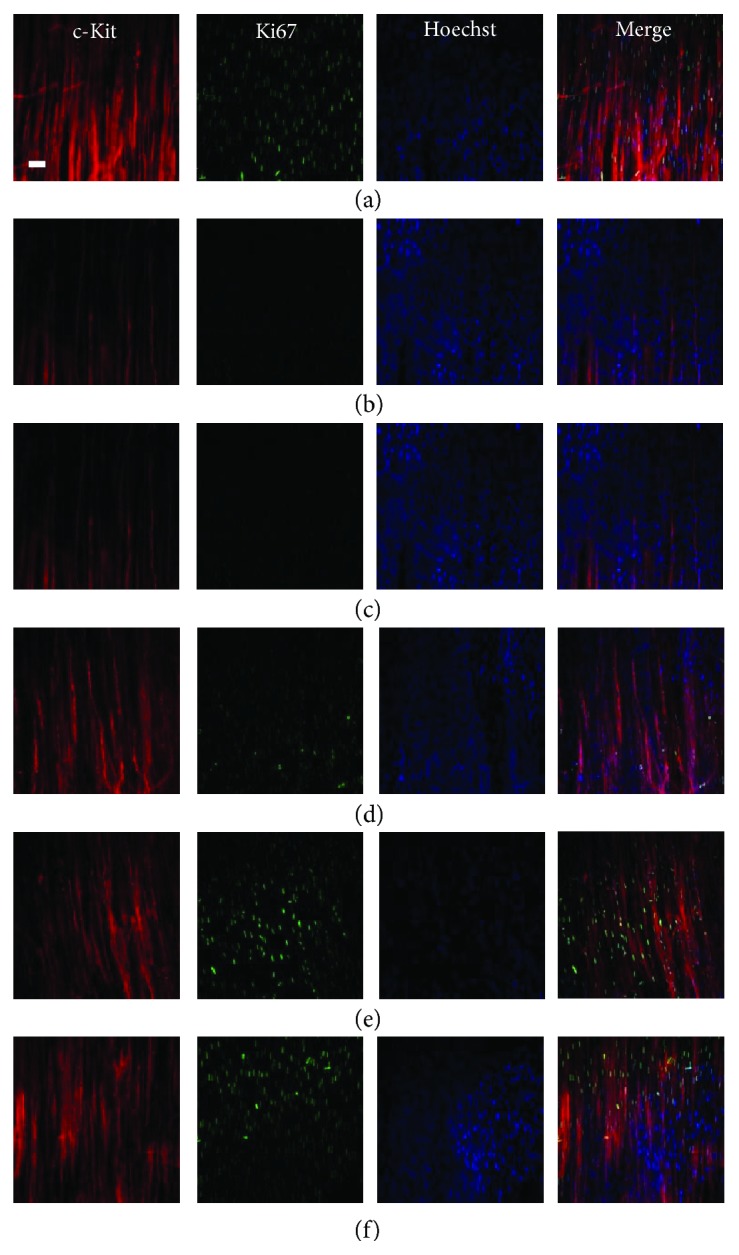
Confocal images of ICC in muscle layers labeled with c-kit (red), Ki67 (green), and Hoechst 33342 (blue). Compared with those in the control group, proliferating ICC were diminished in the DM group and the DM + SGES group. Abundant c-kit/Ki67 double-labeled cells were recognized in the DM + GES1, DM + GES2, and DM + GES3 groups. Scale bars = 20 *μ*m.

The c-kit/Ki67 double-labeled cells were distributed in the control group (Figure 4(a)). As in the DM and DM + SGES groups (Figures 4(b) and 4(c)), no proliferated ICC-IM was observed, accompanied by a considerable reduction in the number of ICC and injured networks. In the DM + GES group (Figures 4(d)–4(f)), increased c-kit+Ki67+ cells were revealed with long slender branches that formed intact networks with c-kit+ cells compared with the DM group.

### 3.4. Detection of SMC

**Figure 5:**
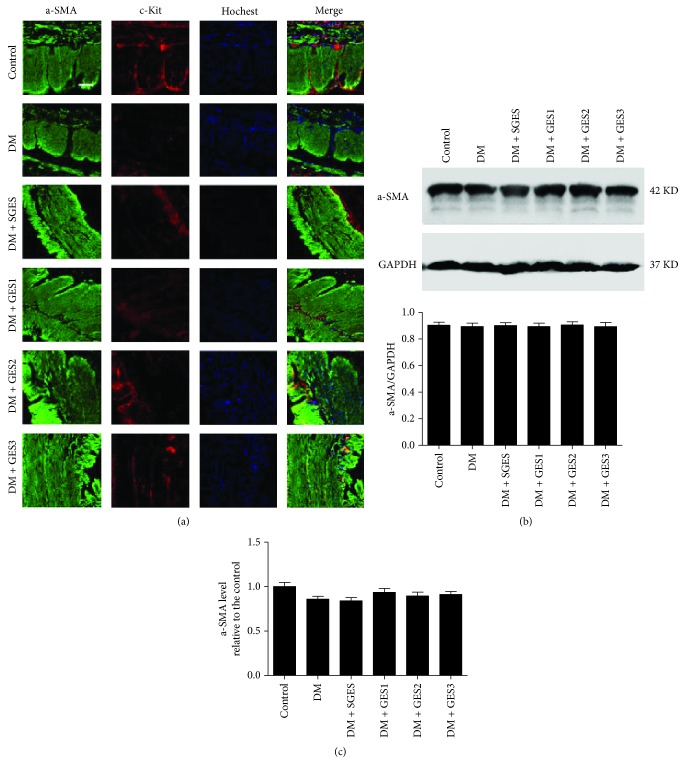
SMC expressed in different groups. (a) Double immunofluorescence showed that after the given gastric electrical stimulation, more c-kit+ cells among a-SMA+ cells were observed, namely, more ICC is distributed in the myenteric layer and the muscle layer (green, a-SMA; red, c-kit; blue, Hoechst 33342; scale bars = 20 *μ*m). (b) Western blot study showed that with long-pulse gastric electrical stimulation, the expression of a-SMA was not significantly changed. (c) RT-PCR showed that the expression of a-SMA mRNA in gastric antrum was not significantly changed in each group.

As shown in Figure 5(a), a lot of a-SMA+ cells with c-kit+ cells were observed in the control group. In the DM and DM + SGES groups, abundant a-SMA+ cells with few c-kit+ cells could be found in the intramuscular layer and the myenteric layer. However, lots of a-SMA+ cells and more c-kit+ cells were observed in the DM + GES groups.

Figure 5(b) shows that there was no significant decrease in the a-SMA expression in the DM group compared with that in the control group (*P* = 0.793). Although different parameters of long-pulse GES were carried out, the expression of a-SMA was not significantly changed (*P* = 1, 0.75, and 0.978).

Further, in Figure 5(c), the mRNA expression of a-SMA of gastric antrum in the DM group was not significantly changed in contrast with that in the control group (*P* = 0.063). Also, there was no significant difference between the DM group and the DM + GES group (*P* = 0.382, 0.988, and 0.091).

### 3.5. GES Contributes to the Ultrastructural Changes of SMC

**Figure 6:**
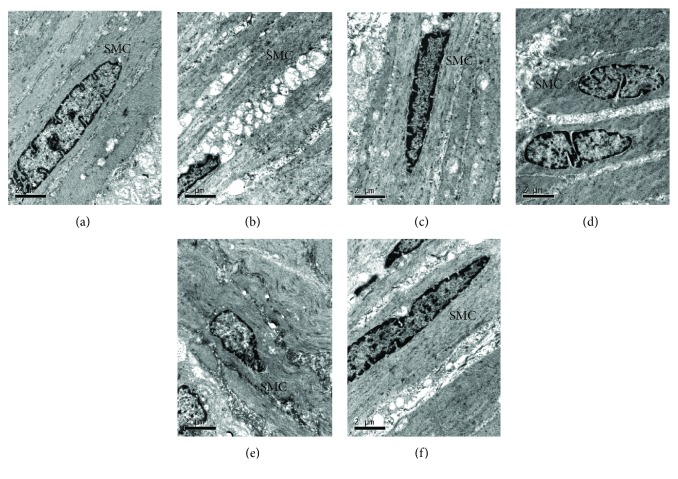
Ultrastructural changes of SMC in the gastric antrum of different groups. In the control group, SMC was spindle shaped with elongated oval nucleus, nuclear uniformed heterochromatin distribution, lots of cytoplasmic dense patches and a dense body, clearly visible filament, and affluent mitochondria. In the DM group, SMC was arranged in disorder with irregular nucleus, massive dark staining chromatin, lots of cytoplasm lysis vacuoles distributed intracellularly, decreased dense bodies and myofilament, swollen mitochondria, vacuolar degeneration, and dissolution. In the DM + GES group, SMC was arranged in neat rows, in fusiform, nuclear heterochromatin distributed evenly, without larger cytoplasmic vacuoles, dense bodies and myofilament rich, and organelles without obvious swelling and expansion. Scale bars are as indicated in each panel.

In the control group (Figure 6(a)), SMC was spindle shaped, the nucleus was elongated oval, nuclear heterochromatin distribution was uniform, cytoplasmic dense patches and a dense body were obviously observed, filament was clearly visible, and the mitochondria were rich. However, in the DM and DM + SGES groups (Figures 6(b) and 6(c)), SMC was arranged in disorder with irregular nucleus, the chromatin was massive with dark staining, many large cytoplasm lysis vacuoles were distributed intracellularly, dense bodies and myofilaments were decreased, and there was swelling in the mitochondria as well as vacuolar degeneration and dissolution. In the DM + GES group (Figures 6(d)–6(f)), SMC was arranged in neat rows, with fusiform, and nuclear heterochromatin was distributed evenly, without larger cytoplasmic vacuoles in the cells, accompanied by dense bodies and rich myofilaments and other organelles.

## 4. Discussion

The results demonstrated that the inhibition of the SCF/c-kit pathway and the damages of ICC and SMC lead to decreased contractile activity of the gastric antrum in diabetic rats. Long-pulse GES could significantly enhance the proliferation of ICC, improve the SCF/c-kit signal pathway, and restore the structure damage of SMC, leading to the improvement in antral contractility.

It has previously been reported that GES has a positive curative effect in the treatment of diabetic gastroparesis [6, 16]. Many studies have demonstrated that short-pulse GES (30–500 *μ*s) improves dyspeptic symptoms, whereas long-pulse GES (100–600 ms) normalizes gastric slow waves and regulates gastric dysrhythmia, but GES with pulse trains (on time and off time for different times) is effective for treating both dyspeptic symptoms and gastric dysrhythmia [17–22]. Li et al. [23] found that gastric pacing (110% of the intrinsic gastric slow wave frequency, 300 ms, 4 mA) could completely entrain the gastric slow wave and normalize gastric dysrhythmias in patients with gastroparesis. Our previous study also showed that long-pulse GES, especially with parameters of 5.5 cpm, 300 ms, and 4 mA, improved the delayed gastric emptying of diabetic rats [24]. Consistent with earlier studies, long-pulse GES could ameliorate the contractile activity of antrum strips in this study. Gastric contractions failed to be enhanced after ICC was destroyed, indicating that ICC played an important role in the contractile activity of gastric muscle strips.

The loss of ICC was certified in the stomach of diabetic gastroparesis experimental models and in patients [25–27]. Many research studies have shown that GES could relieve patients' symptoms of nausea and vomiting, attributed likely to multiple mechanisms involving hormones via the autonomic pathway and enteric glial cells [28, 29]. However, the effects of GES on ICC were scarcely reported. We found that long-pulse GES restored the impaired ICC function by altering the ultrastructure and the number of ICC [15]. Several studies [3, 15, 30, 31] have demonstrated that ICC and SCF were significantly reduced in the gastric antrum of diabetic rats. Transmembrane protein (M-SCF) was described as a more effective ligand for c-kit receptors in our previous study [32]. We also verified that long-pulse GES increased the expression of M-SCF obviously in the gastric antrum of diabetic rats, which was consistent with the earlier report [6]. It was speculated that GES improved gastric contraction, involved in the mechanism of restoring the injury of ICC by promoting the expressions of M-SCF and the SCF/c-kit signaling pathway.

As the number of ICC was an independent factor, the proliferation of ICC-IM was investigated. Previous studies reported that proliferation was an important control factor for ICC recovery when it was lost in adult animals [6, 32]. In our study, the abundant c-kit/Ki67 double-labeled cells were detected in the GES group after a 6-week intervention. Therefore, the proliferation of ICC may be involved in the regulation of GES to restore gastric motility.

SCF is synthesized and secreted by SMC [5, 29]. Diabetic myopathy of skeletal muscle from diabetic models resulted in a significant reduction in the fiber area in eight weeks [33]. A swollen and distended endoplasmic reticulum with an irregular shape was observed in the gastric SMC of diabetic rats for 12 weeks [34]. In our study, we found no obvious reduction in the number of SMC; however, the ultrastructure of SMC was seriously injured in the 6-week diabetes status. It is likely that the damage of SMC gradually appeared with a different duration and different location. Li and Chen [12] have investigated the cellular effects of GES on rat antrum SMC and found that electrical stimulation using a single long pulse and a width of 50 ms or greater was effective in depolarizing cell membrane potentials of SMC. Moreover, Du et al. [35] found that a high-frequency train of stimuli (40 Hz, 10 ms, 150 pA) could invoke and maintain the SMC plateau phase requiring 60% less power and accruing 30% less intracellular Ca^2+^ concentration. Observing the effects of GES on the changes of SMC structure, we found that the injured ultrastructure of SMC was gradually repaired by long-pulse GES, which may promote the synthesis and secretion of SCF.

As an effective treatment and a promising potential choice, GES could be applied in the therapy of gastrointestinal disorders. However, as an invasive treatment, GES needs improvement in its surgical use of electrodes and its clinical application requires additional studies to confirm and optimize its practical application. Different parameters of GES are also closed with the different effects on gastrointestinal motility, so the optimal parameter needs to be further investigated. In summary, long-pulse GES ameliorates the gastric contraction efficiently, depending on the expression of ICC via the proliferation of ICC and depending on the restoration of the SMC structure by the improvement of the SCF/c-kit signal pathway.

## Figures and Tables

**Table 1:** List of designed primers for RT-PCR.

Gene	Primer	5′ → 3′	Size (bp)	GenBank accession no.
*β*-Actin	Sense	CGTTGACATCCGTAAAGACCTC	110	NM_031144
	Antisense	TAGGAGCCAGGGCAGTAATCT		
c-kit	Sense	TGAGAATAAGCAGAGCGAATGG	233	NC_000082
	Antisense	CGTGTATTTGCCCGTGTGAGT		
SCF	Sense	TCCCCACTCTCTTTGGATCTCA	252	NM_021843
	Antisense	GGGCTCACTCCCGAAGAAG		
*α*-SMA	Sense	ATGCTCCCAGGGCTGTTTT	194	AC_000069
	Antisense	CAACCATCACTCCCTGGTGTCT		

## Data Availability

The data used to support the findings of this study are available from the corresponding author upon request.
